# Fluoroquinolone resistance-associated determinants in ciprofloxacin-susceptible *Shigella* isolates from Senegal

**DOI:** 10.3389/fmicb.2026.1974062

**Published:** 2026-10-07

**Authors:** Cheikh Ibrahima Lo, Elhadj Aly Niang, Ousmane Sadio, Safietou Sankhe, Adja Bousso Guèye, Mouhamed Kane, Marie Odile Ndiaye, Maimouna Mbanne, Jaubert Yelkouni, Sokhna Ndongo, Fatime Poulo Ly, Abdoul Aziz Wane, Cheikh Fall, Moussa Moise Diagne, Yakhya Dieye

**Affiliations:** 1 Pôle Microbiologie, Institut Pasteur de Dakar, Dakar, Senegal; 2 École Doctorale des Sciences de la Vie, de la Santé et de l'Environnement, Université Cheikh Anta Diop de Dakar, Dakar, Senegal; 3 Plateforme de Séquençage du Pôle de Virologie, Institut Pasteur de Dakar, Dakar, Senegal; 4 Groupe de Recherche Biotechnologies Appliquées and Bioprocédés Environnementaux (GRBA-BE), École Supérieure Polytechnique, Université Cheikh Anta Diop, Dakar, Senegal

**Keywords:** antimicrobial resistance, ciprofloxacin, fluoroquinolone resistance, gyrA, qnrS1, Senegal, *Shigella*, whole-genome sequencing

## Abstract

**Introduction:**

Fluoroquinolones remain important therapeutic options for shigellosis, but the accumulation of chromosomal and plasmid-mediated resistance determinants threatens their continued effectiveness. Genomic data on fluoroquinolone resistance-associated determinants in *Shigella* from West Africa remain limited. Here, we investigated the epidemiological, phenotypic, and genomic characteristics of clinical *Shigella* isolates recovered through Senegal’s national sentinel surveillance system between 2020 and 2021, with particular emphasis on fluoroquinolone resistance-associated genomic determinants.

**Methods:**

Stool specimens collected from patients with acute diarrhea were cultured for *Shigella*, followed by antimicrobial susceptibility testing and whole-genome sequencing. Genomes were analyzed for antimicrobial resistance determinants, quinolone resistance-determining region (QRDR) mutations, plasmid replicons, virulence-associated genes, sequence types, and core-genome phylogenetic relationships.

**Results:**

Among 900 diarrheal stool specimens, 22 *Shigella* isolates were recovered, including 12 *S. flexneri*, 7 *S. sonnei*, and 3 *S. boydii*. All isolates were phenotypically susceptible to ciprofloxacin, whereas *qnrS1* was detected in 9/22 isolates (40.9%) and the QRDR substitution *gyrA* S83L in 4/22 (18.2%). These determinants occurred in non-overlapping isolates, and no *gyrA* S83V or *parC* QRDR mutation was detected. IncFII replicons were identified in all isolates, while additional replicons showed heterogeneous distributions across species and sequence types. The fluoroquinolone resistance-associated determinants were distributed across distinct genetic backgrounds rather than being restricted to a single lineage.

**Conclusion:**

These findings demonstrate that phenotypic susceptibility to ciprofloxacin may coexist with fluoroquinolone resistance-associated genomic determinants in clinical *Shigella* isolates. Integrating WGS with routine antimicrobial susceptibility testing may strengthen surveillance of emerging fluoroquinolone resistance in *Shigella* in Senegal and West Africa.

## 1 Introduction

Fluoroquinolones remain important broad-spectrum antimicrobials for the treatment of bacterial infections and continue to play a major role in the management of infections caused by several Gram-negative pathogens (Hooper and Jacoby, 2016; Elbehiry et al., 2026; WHO, 2024). However, extensive use of fluoroquinolones in human and veterinary medicine and agriculture has contributed to the emergence and global dissemination of resistant bacteria, threatening the effectiveness of this antimicrobial class (Millanao et al., 2021; Kherroubi et al., 2024). Increasing fluoroquinolone resistance is therefore a major public health concern, affecting both community and healthcare-associated pathogens (EClinicalMedicine, 2021). The dissemination of resistant bacteria and resistance determinants across human, animal, food, and environmental reservoirs further emphasizes the One Health dimension of fluoroquinolone resistance and the need for integrated surveillance strategies (Aslam et al., 2021; Ahmad et al., 2022; Elbehiry et al., 2026).

The development of fluoroquinolone resistance is a stepwise and multifactorial process involving both chromosomal and plasmid-mediated mechanisms. High-level resistance is primarily associated with the accumulation of mutations in the quinolone resistance-determining regions (QRDRs) of genes encoding DNA gyrase and topoisomerase IV, particularly *gyrA* and *parC* (Ruiz, 2003; Jacoby, 2005; Kherroubi et al., 2024). In parallel, plasmid-mediated quinolone resistance (PMQR) determinants, including *qnr* genes, *aac(6′)-Ib-cr, qepA*, and *oqxAB*, can reduce fluoroquinolone susceptibility and facilitate the selection of additional chromosomal mutations under antimicrobial pressure (Jacoby, 2005; Poirel et al., 2012). Changes in membrane permeability and efflux activity may further contribute to reduced susceptibility. Importantly, some of these mechanisms may confer only low-level or incremental resistance and therefore remain undetected by routine phenotypic antimicrobial susceptibility testing, despite potentially contributing to the subsequent development of clinically significant resistance (Hooper and Jacoby, 2016; Kherroubi et al., 2024). The detection of such early genetic changes is consequently important for understanding the evolutionary trajectory of fluoroquinolone resistance.

Conventional antimicrobial susceptibility testing identifies resistance expressed at the time of testing but may not capture genetic determinants associated with early or reduced susceptibility. Whole-genome sequencing (WGS) provides a complementary approach by enabling the simultaneous characterization of acquired resistance genes, chromosomal mutations, plasmid replicons, sequence types, and phylogenetic relationships among bacterial isolates (Didelot et al., 2012; Köser et al., 2012). In particular, WGS can identify resistance-associated genetic determinants in isolates that remain phenotypically susceptible, thereby providing information that may be missed by phenotype-based surveillance alone (Ellington et al., 2017; Hendriksen et al., 2019).

*Shigella* provides an important model for investigating the emergence and evolution of fluoroquinolone resistance. Shigellosis remains a major cause of bacillary dysentery worldwide, particularly among children in low and middle-income countries (LMICs), and continues to contribute substantially to the burden of diarrhoeal disease in sub-Saharan Africa (Kotloff et al., 2013; GBD 2016 Diarrhoeal Disease Collaborators, 2018). The widespread resistance of *Shigella* to historically used antimicrobials, including ampicillin and trimethoprim-sulfamethoxazole, has progressively reduced their therapeutic utility, increasing reliance on alternative agents, including fluoroquinolones when susceptibility is preserved (Livio et al., 2014; WHO, 2005, p. 1). However, the emergence and international dissemination of *Shigella* lineages carrying QRDR mutations and PMQR determinants threaten the continued effectiveness of fluoroquinolones (Chung The et al., 2016; Baker et al., 2023; Kherroubi et al., 2024). Of particular concern, fluoroquinolone resistance-associated determinants may be detected in isolates that remain phenotypically susceptible to ciprofloxacin, suggesting that genomic surveillance can reveal early resistance-associated changes before overt phenotypic resistance becomes apparent.

Despite increasing availability of genomic approaches for antimicrobial resistance surveillance, genomic data on *Shigella* and the early genetic evolution of fluoroquinolone resistance remain limited in West Africa. Senegal has an established sentinel surveillance system for diarrhoeal diseases, providing an important platform for the systematic characterization of circulating enteric pathogens. However, comprehensive investigations combining epidemiological information, antimicrobial susceptibility testing, and whole-genome sequencing of *Shigella* isolates remain scarce (Chattaway et al., 2017; Barry et al., 2026, pp. 2015–2023). Consequently, the distribution of fluoroquinolone resistance-associated determinants, their occurrence across *Shigella* lineages, and their relationship with phenotypic ciprofloxacin susceptibility remain insufficiently characterized in Senegal and the wider West African region.

In this study, we performed an integrated epidemiological and whole-genome analysis of clinical *Shigella* isolates collected through the Senegalese national sentinel surveillance system between 2020 and 2021. We characterized antimicrobial resistance determinants, with particular emphasis on PMQR genes and QRDR mutations associated with fluoroquinolone resistance, and examined their distribution in relation to plasmid replicons, sequence types, and phylogenetic lineages. We specifically investigated whether fluoroquinolone resistance-associated genetic determinants were present among isolates that remained phenotypically susceptible to ciprofloxacin. By integrating antimicrobial susceptibility testing with whole-genome analysis, this study aimed to identify early genomic signatures associated with fluoroquinolone resistance and to assess the potential value of genomic surveillance for detecting resistance-associated changes before phenotypic resistance becomes clinically apparent in *Shigella* circulating in Senegal.

## 2 Materials and methods

### 2.1 Sample collection and processing

Since 2011, the Prevention Department (Epidemiological Surveillance Division) of the Ministry of Health and Social Action (MSAS), in collaboration with the Institut Pasteur de Dakar (IPD), has implemented the Sentinel Syndromic Surveillance System (“4S Network”) for the early detection of priority infectious diseases through a network of sentinel health facilities (Barry et al., 2026, pp. 2015–2023; Faye et al., 2026). The network includes community health centers in decentralized areas across Senegal and three referral hospitals in Dakar.

Between 2020 and 2021, stool samples were collected from patients presenting with acute diarrheal syndrome at participating sentinel sites. All patients presenting with diarrhea were eligible for inclusion. A total of 900 stool specimens were collected during the study period, from which *Shigella* isolates recovered through routine surveillance were included in the present study for phenotypic antimicrobial susceptibility testing and whole-genome sequencing.

### 2.2 Microbiological analysis

Stool specimens were directly inoculated onto Salmonella–Shigella (SS) agar (Oxoid Ltd., Basingstoke, UK) and Hektoen Enteric agar (Liofilchem S.r.l., Roseto degli Abruzzi, Italy), followed by incubation at 37 °C for 18–24 h. Presumptive *Shigella* colonies were subsequently identified by matrix-assisted laser desorption/ionization time-of-flight mass spectrometry (MALDI-TOF MS) (Biotyper® Sirius, Bruker Daltonics, Bremen, Germany).

Confirmed isolates were preserved in glycerol stocks at −80 °C for further analysis. Antimicrobial susceptibility testing (AST) was performed using the disk diffusion method on Mueller–Hinton agar (MHA; Oxoid, Basingstoke, UK). Inhibition zone diameters were measured according to the 2025 CA-SFM/EUCAST clinical breakpoints. The following antimicrobials were tested: amoxicillin (20 μg), ticarcillin (75 μg), amoxicillin–clavulanic acid (20/10 μg), cefoxitin (30 μg), ceftazidime (10 μg), cefepime (30 μg), imipenem (10 μg), ertapenem (10 μg), ciprofloxacin (5 μg), trimethoprim–sulfamethoxazole (1.25/23.75 μg), aztreonam (30 μg), amikacin (30 μg), tobramycin (10 μg), chloramphenicol (30 μg), tetracycline (30 μg), ticarcillin–clavulanic acid (75/10 μg), piperacillin (30 μg), and piperacillin–tazobactam (30/6 μg). AST results were interpreted according to the clinical breakpoints and recommendations of the Comité de l’Antibiogramme de la Société Française de Microbiologie (CA-SFM), 2025.

### 2.3 DNA extraction and whole-genome sequencing

Bacterial genomic DNA was extracted using the QIAamp DNA Mini Kit (QIAGEN, Hilden, Germany) according to the manufacturer’s instructions. DNA concentrations were measured using a Qubit 4 Fluorometer (Thermo Fisher Scientific, Waltham, MA, United States). Sequencing libraries were prepared using the Nextera DNA Flex Library Preparation Kit (Illumina, San Diego, CA, United States) according to the manufacturer’s recommendations and as previously described (Bob et al., 2022). Libraries were indexed, purified, quantified, normalized, and multiplexed. Paired-end sequencing (2 × 150 bp) was performed on an Illumina NextSeq 550 using a 300-cycle High Output kit. The sequencing run containing the isolates analyzed in this study included 85 multiplexed samples and generated an estimated yield of 80.8 Gb, with a cluster density of 105 K/mm^2^, 94.8% of clusters passing filter, and 89.1% of bases having a quality score of Q30 or higher.

### 2.4 Genome assembly and quality control

The quality of raw sequencing reads was assessed using FastQC (v0.11.9). Adapter trimming and filtering of low-quality bases (Phred score < 20) were performed using fastp (v0.20.1). *De novo* genome assembly was conducted using SPAdes (v3.13.1) with default parameters.

### 2.5 Species confirmation and sequence typing

In silico species confirmation was performed using assembled genomes and Mash (v1.1), based on comparison with a locally curated database of complete bacterial chromosome reference genomes retrieved from the NCBI RefSeq database. Multilocus sequence typing (MLST) was performed using the MLST tool (v2.12.0) based on data from the PubMLST database, using the *Shigella* MLST scheme to assign sequence types (STs).

### 2.6 Antimicrobial resistance, virulence, plasmid, and phylogenetic analysis

Antimicrobial resistance genes, virulence factors, and plasmid replicon types were identified using Abricate (v1.0.1). Resistance gene detection was performed using a merged NCBI and ResFinder database, after removal of redundant entries, with thresholds of ≥ 60% sequence coverage and ≥ 90% nucleotide identity. Because Abricate-based acquired resistance gene screening does not identify chromosomal point mutations, ResFinder (v4.6.0) was additionally used to identify resistance-associated point mutations in the QRDRs of *gyrA* and *parC*. Virulence genes were identified using BLASTn against the Virulence Factor Database (VFDB) core dataset which contains experimentally validated virulence factors. Hits were retained when sequence identity and gene coverage were ≥70%, with an E-value ≤1 × 10^−5^. Presence–absence matrices of virulence genes were generated and used for hierarchical clustering and comparative analyses.

Phylogenetic relationships among isolates were inferred based on core-genome single-nucleotide polymorphisms (SNPs) identified using Snippy (v4.5.0). A maximum-likelihood phylogenetic tree was constructed using IQ-TREE (v2.4.0) with 1,000 bootstrap replicates, and recombination was assessed and filtered using Gubbins (v3.1.6). Pairwise core-genome SNP distances were calculated from the resulting SNP matrix to quantify genomic variation among isolates within the same sequence type. All analyses, except phylogenetic tree reconstruction, were integrated into a locally developed bioinformatics pipeline.

### 2.7 Epidemiological and statistical analysis

Demographic and epidemiological variables, including age, sex, and geographic location, were extracted from the surveillance database. Continuous variables were summarized using the median and interquartile range (IQR), while categorical variables were expressed as frequencies and percentages.

### 2.8 Ethical considerations

This study was conducted as part of the national sentinel surveillance program coordinated by the Ministry of Health and Social Action of Senegal in collaboration with the Institut Pasteur de Dakar. The study protocol was reviewed and approved by the Comité National d’Ethique pour la Recherche en Santé (reference number: SEN19/52). All samples were anonymized prior to analysis, and informed consent was obtained in accordance with national regulations.

## 3 Results

### 3.1 Epidemiological and species characteristics of *Shigella* isolates

Between 2020 and 2021, 900 diarrheal stool specimens were collected from patients attending sentinel surveillance sites across Senegal. A total of 22 *Shigella* isolates (2.44%) were recovered by culture-based methods and identified at the species level using MALDI-TOF MS.

The 22 isolates corresponded to 22 patients. Females accounted for 59.1% of cases (13/22), while males accounted for 40.9% (9/22). The median age was 3.6 years (range, 1.3–67 years) (IQR, 2.7–9.6), and children under five years of age represented 63.6% of cases (14/22) (Table S1).

The 22 genomes had a mean sequencing depth of 155.6 × (range, 119–180×) (Lo et al., 2026). Among the 22 isolates, *Shigella flexneri* was the predominant species (12/22, 54.5%), followed by *Shigella sonnei* (7/22, 31.8%) and *Shigella boydii* (3/22, 13.6%). The geographic distribution of the isolates and their corresponding sequence types across Senegal is presented in Figure 1.

**Figure 1:**
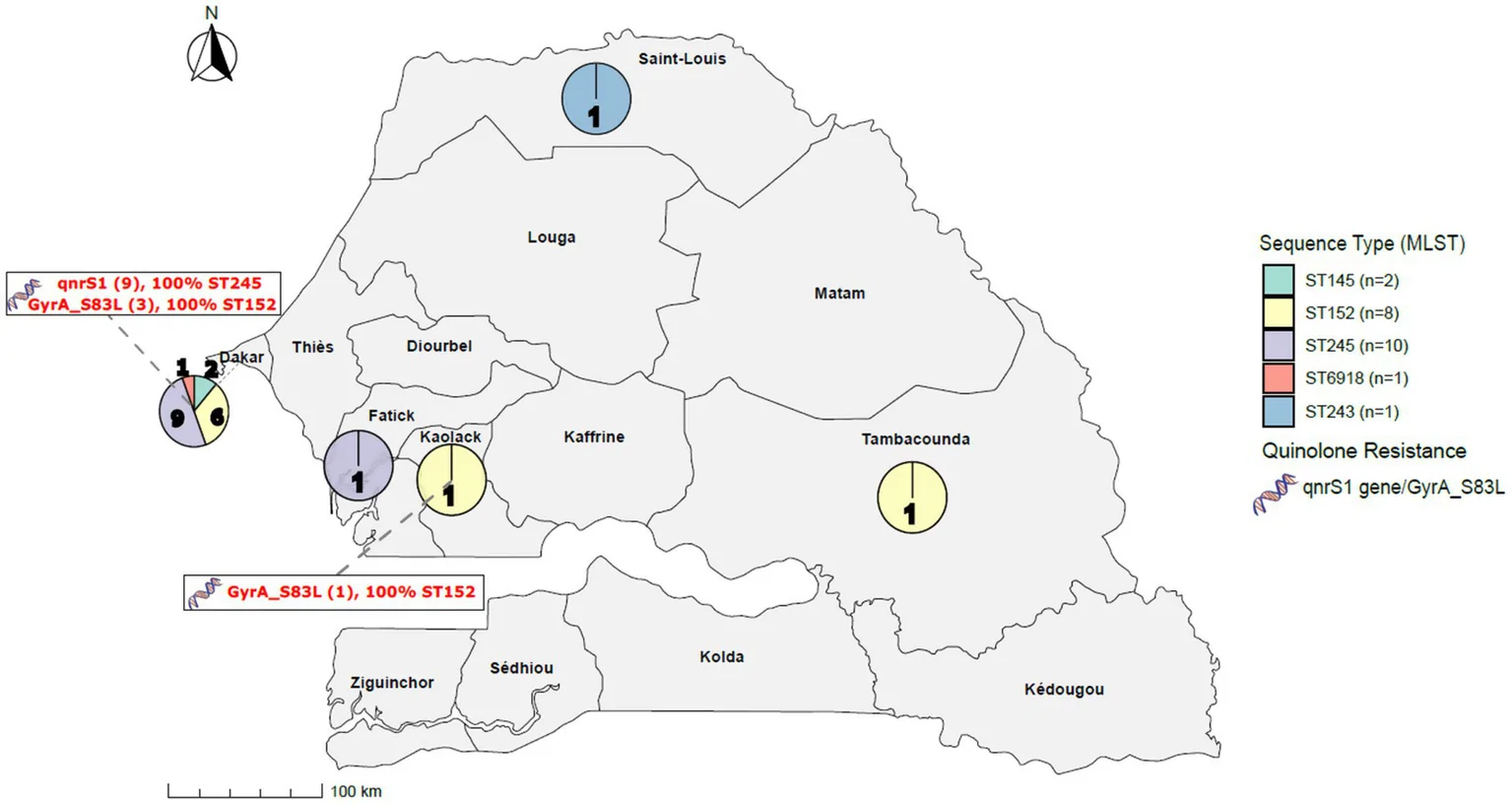
Geographic distribution of the 22 *Shigella* isolates, sequence types, and fluoroquinolone resistance-associated determinants in Senegal. Pie charts indicate the sequence-type composition of isolates recovered at each sampling location. The colors correspond to the sequence types shown in the legend. The distribution of *qnrS1* and *gyrA* S83L is indicated by the corresponding annotations.

### 3.2 Antimicrobial susceptibility profiles and genotype–phenotype concordance

Antimicrobial susceptibility testing revealed heterogeneous resistance profiles among the 22 *Shigella* isolates (Table 1). The highest level of resistance was observed for trimethoprim–sulfamethoxazole, with 21 of 22 isolates (95.5%) classified as resistant. This phenotype was supported by the widespread detection of antifolate resistance determinants, including *dfrA1, dfrA14, sul1*, and *sul2*, indicating a high level of genotype–phenotype concordance for this antibiotic class. Resistance to amoxicillin, ticarcillin, and piperacillin was observed in 11 isolates (50.0%) and was associated with the presence of *blaTEM-1* and *blaEC*. In contrast, all isolates remained susceptible to *β*-lactam/β-lactamase inhibitor combinations, cephalosporins, monobactams, and carbapenems, and no extended-spectrum *β*-lactamase or carbapenemase genes were identified.

**Table 1:** Genotype–phenotype concordance of antimicrobial resistance determinants among *Shigella* isolates from Senegal.

Antimicrobial class	Phenotypic profile (AST)	Genomic determinants detected (WGS)	Genotype–phenotype interpretation
Penicillins	AMX, TIC and PIP resistance in 11/22 (50.0%)	*blaTEM-1*, *blaEC*	Partial concordance
β-lactam/β-lactamase inhibitor combinations	All isolates susceptible to AMC, TIM and TZP (22/22)	*blaTEM-1* detected in several isolates	Genotype without phenotype
Cephalosporins and monobactam	All isolates susceptible to FOX, CAZ, FEP and ATM (22/22)	No ESBL-associated genes detected	Concordant
Carbapenems	All isolates susceptible to ETP and IPM (22/22)	No carbapenemase genes detected	Concordant
Aminoglycosides	All isolates susceptible to TOB and AMK (22/22)	*aph(3″)-Ib*, *aph(6)-Id*, *aadA1*, *sat2*	Genotype without phenotype
Fluoroquinolones	All isolates susceptible to CIP (22/22)	*qnrS1*, *gyrA* S83L	Genotype without phenotype
Antifolates	SXT resistance in 21/22 (95.5%)	*dfrA1*, *dfrA14*, *sul1*, *sul2*	Strong genotype–phenotype concordance
Tetracyclines	TET resistance in 18/22 (81.8%)	*tet(A)* and/or *tet(B)* detected in 20/22 (90.9%)	High concordance; discordance in 2 isolates
Amphenicols	All isolates susceptible to CHL (22/22)	*catA1* detected in 1 isolate	Genotype without phenotype

AST, antimicrobial susceptibility testing; WGS, whole-genome sequencing; AMX, amoxicillin; AMC, amoxicillin–clavulanic acid; TIC, ticarcillin; TIM, ticarcillin–clavulanic acid; PIP, piperacillin; TZP, piperacillin–tazobactam; FOX, cefoxitin; CAZ, ceftazidime; FEP, cefepime; ATM, aztreonam; ETP, ertapenem; IPM, imipenem; TOB, tobramycin; AMK, amikacin; CIP, ciprofloxacin; SXT, trimethoprim-sulfamethoxazole; TET, tetracycline; CHL, chloramphenicol.

Genotype without phenotype indicates detection of a resistance-associated determinant in an isolate that remained phenotypically susceptible to the corresponding antimicrobial. Concordant indicates agreement between the observed phenotype and the detected genomic determinants.

Resistance to tetracycline was observed in 18/22 isolates (81.8%). Corresponding resistance determinants, *tet(A)* and/or *tet(B)*, were detected in 20/22 isolates (90.9%), including two isolates that remained phenotypically susceptible to tetracycline.

All isolates were susceptible to ciprofloxacin, tobramycin, and amikacin. Aminoglycoside resistance-associated genes, including *aph(3″)-Ib, aph(6)-Id, aadA1*, and *sat2*, were detected despite the absence of phenotypic resistance to aminoglycosides, indicating additional instances of genotype–phenotype discordance.

### 3.3 Fluoroquinolone resistance-associated genomic determinants

Despite universal phenotypic susceptibility to ciprofloxacin, whole-genome sequencing identified fluoroquinolone resistance-associated determinants in 13 of the 22 *Shigella* isolates (59.1%). Our Table 2 shows the plasmid-mediated quinolone resistance gene *qnrS1* was detected in 9/22 isolates (40.9%), while the quinolone resistance-determining region (QRDR) substitution *gyrA* S83L was identified in 4/22 isolates (18.2%). No *gyrA* S83V substitution or *parC* QRDR mutation was detected.

**Table 2:** Genomic characteristics of *Shigella* isolates carrying fluoroquinolone resistance-associated determinants.

Species (ST)	No. isolates	FQ determinant	CIP phenotype	Plasmid replicon profiles
*S. flexneri* (ST245)	9	*qnrS1*	S (9/9)	IncFII + IncFIB(K) (5); IncFII + Col156 (1); IncFII + IncFIB(K) + Col(BS512) (1); IncFII + Col(BS512) + Col(MG828) (1); IncFII + IncFIB(K) + Col(MG828) (1)
*S. sonnei* (ST152)	4	*gyrA* S83L	S (4/4)	IncFII + Col156 + Col(BS512) (3); IncFII + Col156 + Col(MG828) (1)

CIP, ciprofloxacin; S, susceptible; ST, sequence type. All 13 isolates carrying qnrS1 or gyrA S83L were phenotypically susceptible to ciprofloxacin. No isolate carried both determinants. Plasmid replicon profiles represent replicons detected in the corresponding isolates and do not establish physical localization of resistance determinants on specific plasmids.

All isolates carrying *qnrS1* or *gyrA* S83L remained susceptible to ciprofloxacin, resulting in a genotype–phenotype discordance under the applied clinical breakpoints.

### 3.4 Plasmid replicon profiles and associated antimicrobial resistance determinants

Plasmid replicon analysis identified a diverse plasmid repertoire among the 22 *Shigella* isolates (Table 3). IncFII replicons were detected in all isolates (22/22, 100%) and were present across *S. flexneri*, *S. sonnei*, and *S. boydii*. IncFIB (K), Col(BS512), Col156, and Col(MG828) were detected in 10 (45.5%), 9 (40.9%), 8 (36.4%), and 5 (22.7%) isolates, respectively, while IncQ1, IncY, IncI1 (Alpha), Col440I, and IncFIB(AP001918) were each detected in one isolate (4.5%).

**Table 3:** Plasmid replicon profiles and co-occurring antimicrobial resistance determinants among *Shigella* isolates (*n* = 22).

Plasmid replicon type	No. of isolates (n)	Percentage (%)	*Shigella* species involved	AMR determinants co-occurring in plasmid-positive isolates
IncFII (all variants)	22	100.0	*S. flexneri* (12), *S. sonnei* (7), *S. boydii* (3)	*blaTEM-1*, *blaEC*, *dfrA1*, *dfrA14*, *sul1*, *sul2*, *tet(A)*, *tet(B)*, *qnrS1*
IncFIB (K)	10	45.5	*S. flexneri* (7), *S. sonnei* (2), *S. boydii* (1)	*blaTEM-1*, *blaEC*, *dfrA1*, *dfrA14*, *sul2*, *tet(A)*, *tet(B)*, *qnrS1*
Col (BS512) (all variants)	9	40.9	*S. flexneri* (4), *S. sonnei* (4), *S. boydii* (1)	*blaTEM-1*, *blaEC*, *dfrA1*, *dfrA14*, *sul2*, *tet(A)*, *tet(B)*, *qnrS1*, *gyrA* S83L
Col156	8	36.4	*S. sonnei* (5), *S. boydii* (2), *S. flexneri* (1)	*blaTEM-1*, *blaEC*, *dfrA1*, *dfrA14*, *sul2*, *tet(A)*, *gyrA* S83L
Col (MG828)	5	22.7	*S. sonnei* (3), *S. flexneri* (2)	*blaTEM-1*, *blaEC*, *dfrA1*, *dfrA14*, *sul2*, *tet(A)*, *qnrS1*, *gyrA* S83L
IncQ1	1	4.5	*S. sonnei* (1)	*aadA1*, *dfrA1*, *sul2*, *tet(A)*
IncY	1	4.5	*S. flexneri* (1)	*blaTEM-1*, *catA1*, *dfrA14*, *sul1*, *sul2*, *tet(A)*, *tet(B)*
IncI1 (Alpha)	1	4.5	*S. flexneri* (1)	*blaTEM-1*, *blaEC*, *dfrA14*, *sul2*, *tet(A)*
Col440I	1	4.5	*S. sonnei* (1)	*aadA1*, *dfrA1*, *sul2*, *tet(A)*
IncFIB (AP001918)	1	4.5	*S. sonnei* (1)	*aadA1*, *dfrA1*, *sul2*, *tet(A)*

Plasmid replicon types were identified by whole-genome sequencing. Percentages were calculated using the total number of isolates included in the study (*n* = 22). “All variants” refers to multiple allelic variants detected within the same incompatibility group. Antimicrobial resistance determinants listed represent genes detected in isolates carrying the corresponding plasmid replicon and do not imply physical localization of these genes on the identified plasmid.

Multiple antimicrobial resistance determinants co-occurred with several plasmid replicon types. In particular, *qnrS1* was detected among isolates carrying IncFII, IncFIB (K), Col(BS512), and Col(MG828) replicons, while *gyrA* S83L was identified in isolates carrying Col(BS512), Col156, and Col(MG828). Several isolates harbored multiple plasmid replicons simultaneously (Table 3).

### 3.5 Phylogenomic structure and distribution of resistance determinants

To place the Senegalese isolates in a broader context, comparative core-genome phylogenies were constructed using publicly available *Shigella* genomes from Africa and other geographic regions for *S. flexneri*, *S. sonnei*, and *S. boydii* (Figures 2A–C). The Senegalese isolates were distributed within the corresponding phylogenetic diversity, with ST245 predominating among *S. flexneri* and ST152 among *S. sonnei*.

**Figure 2:**
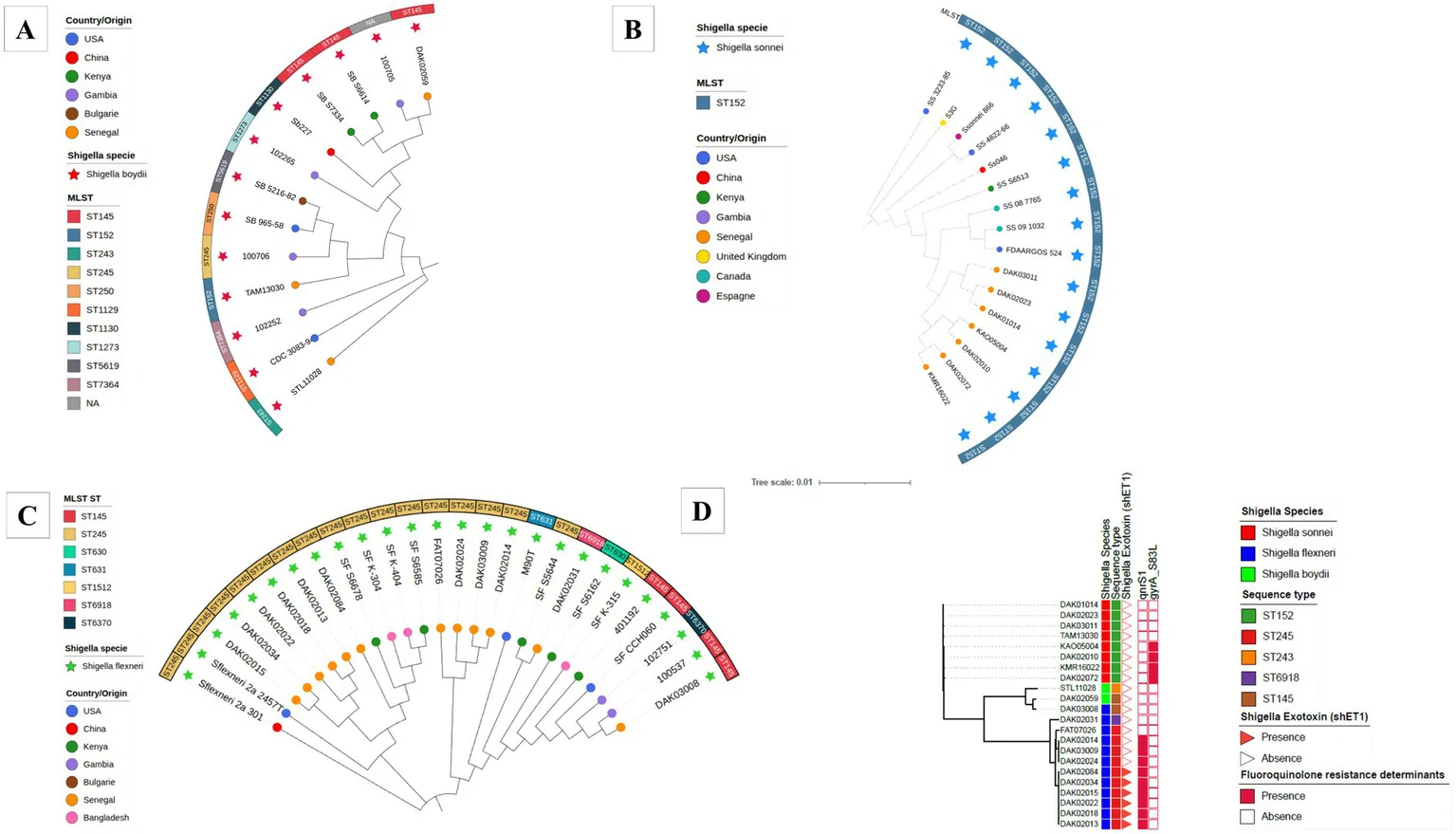
Phylogenomic positioning of Senegalese *Shigella* isolates within global *S. flexneri*, *S. sonnei*, and *S. boydii* populations. Circular core-genome phylogenies of **(A)** *S. boydii*, **(B)** *S. sonnei*, and **(C)** *S. flexneri*, including Senegalese isolates and publicly available genomes from Africa and other geographic regions. Metadata tracks indicate species, MLST, and country of origin. **(D)** Core-genome phylogeny of the 22 Senegalese *Shigella* isolates with associated species, sequence type (ST), *Shigella* enterotoxin 1 (*shET1*), and fluoroquinolone resistance-associated determinants (*qnrS1* and *gyrA* S83L). Senegalese isolates are highlighted in red. The tree scale represents substitutions per site.

Within the Senegalese dataset, cgSNP-based phylogenetic analysis resolved the 22 *Shigella* isolates into distinct clusters largely corresponding to species and STs (Figure 2D). Pairwise core-genome SNP analysis further quantified genomic variation within shared STs (Table 4). Among the ten *S. flexneri* ST245 isolates, distances ranged from 1 to 1,708 SNPs (median, 1,677), while the seven *S. sonnei* ST152 isolates showed distances ranging from 54 to 393 SNPs (median, 334).

**Table 4:** Pairwise core-genome SNP distances among Senegalese *Shigella* isolates within the same sequence type.

Species	ST	No. isolates	Strain numbers	No. pairwise comparisons	Core-SNP distance, min–max	Median
*S. flexneri*	ST245	10	DAK02013, DAK02014, DAK02015, DAK02018, DAK02022, DAK02024, DAK02034, DAK02084, DAK03009, FAT07026	45	1–1,708	1,677
*S. sonnei*	ST152	7	DAK01014, DAK02010, DAK02023, DAK02072, DAK03011, KAO05004, KMR16022	21	54–393	334
*S. flexneri*	ST145	1	DAK03008	—	NA	NA
*S. flexneri*	ST6918	1	DAK02031	—	NA	NA
*S. boydii*	ST145	1	DAK02059	—	NA	NA
*S. boydii*	ST243	1	STL11028	—	NA	NA
*S. boydii*	ST152	1	TAM13030	—	NA	NA

Core-SNP distances represent pairwise differences among isolates belonging to the same sequence type. Median values were calculated from all pairwise comparisons within each ST. NA, not applicable because only one isolate was available for the corresponding ST.

The distribution of fluoroquinolone resistance-associated determinants was heterogeneous. *qnrS1* was detected in 9 isolates, whereas *gyrA* S83L was detected in 4 isolates; the two determinants occurred in non-overlapping isolates and were not confined to a single phylogenetic cluster (Figure 2D). Overall, the phylogenomic analysis demonstrated the coexistence of multiple *Shigella* lineages and heterogeneous distributions of fluoroquinolone resistance-associated determinants across the study population.

### 3.6 Virulence-associated gene profiles

Virulence gene profiling revealed a largely conserved repertoire of *Shigella*-associated determinants across the 22 isolates (Figure 3). Genes associated with the invasion plasmid and type III secretion system (T3SS), including *virF, virB, virA, ipaA-D*, *ipg*, *mxi*, and *spa* operon components, were widely distributed among the isolates. The *ipaH* gene family was detected in nearly all isolates, further supporting the conversation of major virulence-associated determinants across the study population.

**Figure 3:**
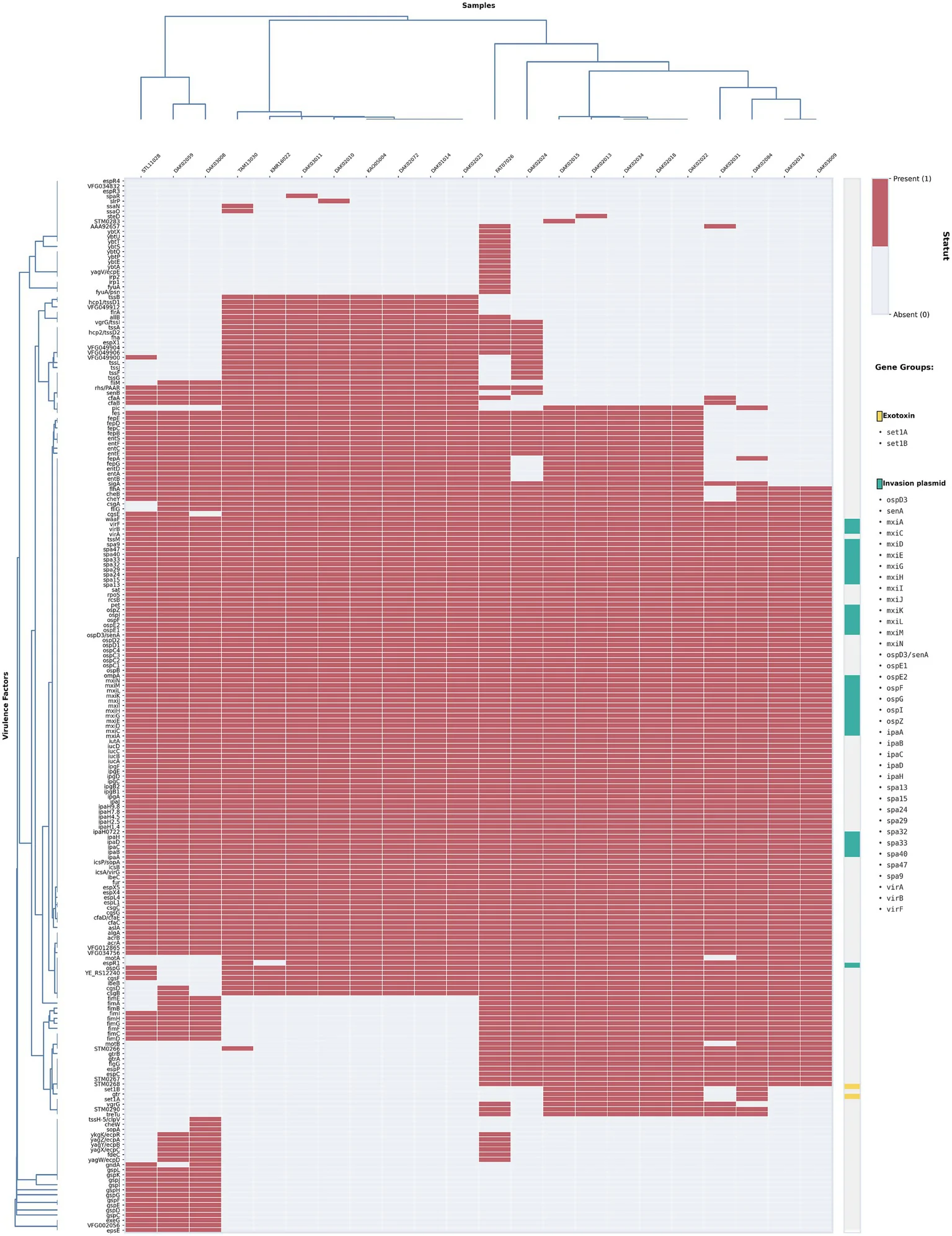
Virulence-associated gene profiles of *Shigella* isolates. Virulence-associated genes (rows) and *Shigella* isolates (columns) were hierarchically clustered according to their presence–absence profiles. The heatmap illustrates the distribution of major virulence-associated determinants, including invasion plasmid-associated genes, type III secretion system (T3SS) components, and exotoxin-associated genes. Red indicates gene presence and white indicates gene absence. Dendrograms represent similarities in virulence gene content among isolates.

Hierarchical clustering of virulence gene presence-absence profiles identified groups of isolates with similar virulence repertoires, broadly reflecting the genetic relatedness observed among the isolates. Most of the observed variation involved accessory virulence-associated genes rather than the major invasion and T3SS components. Notably, *shET1* was detected in 6/22 isolates (27.3%), all of which belonged to *S. flexneri* ST245, and was not detected among the *S. sonnei* or *S. boydii* isolates. Thus, although the core virulence repertoire was largely conserved, specific accessory virulence determinants showed lineage-associated distributions.

### 3.7 Discussion

#### 3.7.1 Epidemiological and genomic context of *Shigella* in Senegal

The epidemiological profile of the isolates in this study is broadly consistent with the distribution of shigellosis reported across sub-Saharan Africa (Stenhouse et al., 2023; Nyarkoh et al., 2024; Somda et al., 2025). The predominance of *S. flexneri* (54.5%), followed by *S. sonnei* (31.8%) and *S. boydii* (13.6%), is in agreement with recent African evidence showing that *S. flexneri* remains the predominant *Shigella* species in the region (Kasumba et al., 2023; Minawir et al., 2026; Mwendera et al., 2026). In 2024 a systematic review and meta-analysis including 116 studies from 29 African countries estimated that *S. flexneri* accounted for 53.6% of reported *Shigella* isolates, while *S. sonnei* represented 11.5%, although substantial geographic heterogeneity was observed (Nyarkoh et al., 2024).

The clustering of cases among young children is also consistent with the recognized epidemiology of shigellosis in low and middle-income countries. Recent African studies continue to identify children under 5 years of age as a major affected population, although estimates vary according to diagnostic methods, surveillance design, and geographical setting (Stenhouse et al., 2023; Yousafzai et al., 2026). The relatively low proportion of culture-confirmed *Shigella* in our study should therefore be interpreted in the context of a culture-based surveillance approach, as molecular diagnostic methods generally provide higher detection rates (Thiem et al., 2004; Liu et al., 2024).

Beyond these epidemiological observations, the identification of multiple *Shigella* sequence types among the Senegalese isolates indicates that the circulating population was genetically heterogeneous rather than dominated by a single lineage (Bengtsson et al., 2022; Stenhouse et al., 2023; Ayele et al., 2024). This finding is consistent with genomic studies from other African settings, including South Africa, where whole-genome analysis revealed substantial lineage diversity and differences in antimicrobial resistance and virulence profiles among circulating *Shigella* populations (Stenhouse et al., 2023). In Senegal, where genomic data on *Shigella* have remained limited, these findings provide an initial genomic framework for linking bacterial population structure with antimicrobial resistance determinants and support the integration of WGS into routine enteric pathogen surveillance.

#### 3.7.2 Fluoroquinolone resistance-associated determinants in ciprofloxacin-susceptible isolates

The most important finding of this study was the detection of fluoroquinolone resistance-associated genomic determinants in ciprofloxacin-susceptible *Shigella* isolates. Although all 22 isolates remained phenotypically susceptible to ciprofloxacin, 9 (40.9%) carried *qnrS1* and 4 (18.2%) carried the QRDR substitution *gyrA* S83L. These determinants were found in non-overlapping isolates, and no *gyrA* S83V or *parC* QRDR mutation was detected. Thus, 13/22 isolates (59.1%) carried at least one fluoroquinolone resistance-associated determinant despite remaining susceptible to ciprofloxacin under the applied clinical breakpoints. This type of genotype–phenotype discordance has been previously reported in *Shigella* and other *Enterobacterales*, where single quinolone resistance determinants may not translate into clinical resistance but may indicate reduced susceptibility or an early stage of resistance evolution (Hooper and Jacoby, 2016; Partridge et al., 2018; Kalinen et al., 2025).

This apparent genotype–phenotype discordance is consistent with the stepwise nature of fluoroquinolone resistance development in *Shigella*. Individual quinolone resistance determinants may reduce antimicrobial susceptibility without necessarily exceeding clinical resistance breakpoints. In particular, *qnr* genes encode pentapeptide repeat proteins that protect DNA gyrase and topoisomerase IV from quinolone-mediated inhibition, thereby conferring a low-level resistance background that can facilitate the selection of additional chromosomal mutations (Hooper and Jacoby, 2016). Similarly, the *gyrA* S83L substitution is a well-characterised QRDR alteration associated with reduced fluoroquinolone susceptibility in *Shigella* (Chung The et al., 2019; Yu et al., 2020). Previous studies have shown that single *gyrA* S83L substitutions have been associated with reduced fluoroquinolone susceptibility, whereas higher levels of resistance are generally associated with the accumulation of additional QRDR mutations and/or other resistance mechanisms (Azmi et al., 2014).

#### 3.7.3 Plasmid and phylogenomic context of fluoroquinolone resistance-associated determinants

The plasmid and phylogenomic analyses provided additional context for the fluoroquinolone resistance-associated determinants identified in this study. IncFII was detected in all 22 isolates, indicating that this replicon group was a common component of the plasmid repertoire of the Senegalese *Shigella* population. Other replicons, including IncFIB(K), Col(BS512), Col156, and Col(MG828), occurred at lower frequencies and were distributed across different *Shigella* species. The frequent detection of IncFII is consistent with the importance of IncF-related plasmid backbones in *Shigella* and other *Enterobacterales*, where plasmids can contribute to the acquisition and maintenance of multiple antimicrobial resistance determinants (Rozwandowicz et al., 2018; Muthuirulandi Sethuvel et al., 2019).

Of particular interest, *qnrS1* was detected in isolates carrying several plasmid replicon types, including IncFII, IncFIB(K), Col(BS512), and Col(MG828), whereas *gyrA* S83L occurred in isolates with Col(BS512), Col156, and Col(MG828). This diversity of plasmid backgrounds contrasts with recent genomic observations from Zambia, where *qnrS1* was found to be carried exclusively on an IncFIB(K) plasmid among the *Shigella* isolates analyzed (Chibuye et al., 2026). Similarly, genomic analysis of *Shigella* from Malawi identified a multidrug-resistant plasmid carrying *qnrS1*, with highly similar plasmid sequences detected in different *S. flexneri* serotypes, supporting the potential for dissemination of PMQR-associated plasmids across distinct bacterial lineages (Stenhouse et al., 2021). These comparisons suggest that the plasmid context of *qnrS1* may vary geographically and among *Shigella* populations.

However, the co-occurrence of a resistance determinant and a plasmid replicon within the same isolate does not by itself demonstrate that the determinant is physically located on that plasmid. Therefore, our data support an association between *qnrS1* and specific plasmid backgrounds but do not establish its physical plasmid localization or horizontal transfer. This distinction is particularly important for *gyrA* S83L, which represents a chromosomal QRDR mutation and should therefore not be interpreted as a plasmid-mediated resistance determinant (Yu et al., 2020). Complete plasmid reconstruction, ideally using long-read sequencing, would be required to resolve these associations.

The phylogenomic analysis further showed that *qnrS1* and *gyrA* S83L were distributed across different genetic backgrounds rather than being restricted to a single dominant lineage. This observation is consistent with recent global genomic analyses of PMQR-positive *Shigella*, which identified *qnrS* as the predominant PMQR determinant and detected PMQR-positive isolates across multiple sequence types, with ST152 and ST245 representing major PMQR-associated lineages (Geng et al., 2026). In our collection, the non-overlapping distribution of *qnrS1* and *gyrA* S83L across the phylogeny may reflect differences in genetic background or resistance acquisition. However, the limited number of isolates and cross-sectional design prevent us from distinguishing between clonal inheritance, independent acquisition of resistance determinants, or, for *qnrS1*, plasmid-mediated dissemination.

#### 3.7.4 Antimicrobial resistance patterns and genotype–phenotype concordance

Beyond fluoroquinolone-associated determinants, the antimicrobial resistance profiles observed in this study revealed marked differences in the relationship between genotype and phenotype across antibiotic classes. Trimethoprim-sulfamethoxazole showed the highest phenotypic resistance rate in our collection, with 21/22 isolates (95.5%) classified as resistant, and this phenotype was accompanied by a broad distribution of *dfrA* and *sul* resistance determinants. This high genotype–phenotype concordance is consistent with the substantial burden of trimethoprim-sulfamethoxazole resistance reported among *Shigella* isolates in Africa, where resistance to this older antimicrobial has remained widespread (Nyarkoh et al., 2024).

In contrast, all isolates remained susceptible to aminoglycosides despite the detection of several aminoglycoside resistance-associated genes, including *aph(3″)-Ib*, *aph(6)-Id*, *aadA1*, and *sat2*. This discordance illustrates that the detection of a resistance-associated gene does not necessarily translate into a resistant phenotype, as gene expression, gene functionality, bacterial genetic background, and the magnitude of resistance conferred by individual determinants may influence phenotypic susceptibility (Strahilevitz et al., 2009; Yu et al., 2020). The contrasting patterns observed for trimethoprim–sulfamethoxazole and ciprofloxacin further illustrate the variable relationship between resistance genotype and phenotype: resistance to trimethoprim–sulfamethoxazole was both phenotypically expressed and genomically supported, whereas *qnrS1* and *gyrA* S83L were detected in ciprofloxacin-susceptible isolates. These findings support the complementary use of WGS and phenotypic antimicrobial susceptibility testing, as genomic surveillance may identify resistance-associated determinants not yet reflected in routine susceptibility categories, while phenotypic testing remains essential for assessing the current antimicrobial susceptibility of clinical isolates (Sadouki et al., 2017; Kong et al., 2023).

#### 3.7.5 Strengths and limitations

This study integrates phenotypic antimicrobial susceptibility testing with whole-genome sequencing, enabling the characterization of resistance determinants, QRDR mutations, plasmid replicons, virulence-associated genes, sequence types, and phylogenomic relationships. This integrated approach was particularly valuable for identifying fluoroquinolone resistance-associated determinants in isolates that remained phenotypically susceptible to ciprofloxacin. In addition, the use of isolates collected through the Senegalese sentinel surveillance system provides clinically relevant genomic data from a setting where *Shigella* genomic surveillance remains limited.

The small sample size (*n* = 22) limits comparisons between species, sequence types, and resistance-associated profiles, and the findings should not be interpreted as national prevalence estimates. The cross-sectional sampling period (2020–2021) also precludes assessment of temporal trends in fluoroquinolone resistance. Finally, the use of Illumina short-read sequencing limited the resolution of plasmid architecture and genomic localization. Future studies using long-read sequencing and functional approaches will be necessary to further characterize their genomic context and dissemination potential.

### 3.8 Conclusion

This study provides a genomic characterization of *Shigella* isolates from Senegal and highlights a substantial gap between fluoroquinolone resistance-associated genotypes and ciprofloxacin susceptibility. Although all isolates were phenotypically susceptible to ciprofloxacin, almost 60% carried *qnrS1* and/or *gyrA* S83L. These findings underscore the added value of integrating WGS with phenotypic antimicrobial susceptibility testing for the detection and surveillance of emerging fluoroquinolone resistance in *Shigella* in Senegal and West Africa.

## Data Availability

The datasets presented in this study can be found in online repositories. The names of the repository/repositories and accession number(s) can be found in the article/Supplementary material.
