# Supplementary material for: Fluoroquinolone resistance-associated determinants in ciprofloxacin-susceptible Shigella isolates from Senegal
Source: Front Microbiol. 2026 Oct 7;17:1974062. doi: 10.3389/fmicb.2026.1974062 (PMC13683774; doi:10.3389/fmicb.2026.1974062)
Supplement: Supplementary file 1 [file Table_1.DOCX]

Supplementary Material

# 1 Supplementary Data

## 1.1 Supplementary Tables

| **Strain ID** | **Region** | **Sample_type** | **Age** | **Sexe** | **Species** | **Genome size (bp)** | **G+C%** | **Coverage (x)** | **ST** | **Accession Numbers** |
| --- | --- | --- | --- | --- | --- | --- | --- | --- | --- | --- |
| DAK02059 | Dakar | Human Stool | 2 | F | *Shigella boydii* | 4,376,029 | 50.63 | 168 | 145 | JBSVEA000000000 |
| DAK02013 | Dakar | Human Stool | 67 | F | *Shigella flexneri* | 4,512,244 | 50.51 | 180 | 245 | JBSVDR000000000 |
| DAK02014 | Dakar | Human Stool | 3.7 | M | *Shigella flexneri* | 4,468,602 | 50.48 | 170 | 245 | JBSVDS000000000 |
| DAK02015 | Dakar | Human Stool | 1.3 | M | *Shigella flexneri* | 4,514,973 | 50.50 | 154 | 245 | JBSVDT000000000 |
| DAK02018 | Dakar | Human Stool | 4.6 | F | *Shigella flexneri* | 4,504,314 | 50.51 | 172 | 245 | JBSVDU000000000 |
| DAK02022 | Dakar | Human Stool | 2.9 | F | *Shigella flexneri* | 4,511,697 | 50.50 | 149 | 245 | JBSVDV000000000 |
| DAK02024 | Dakar | Human Stool | 3.4 | M | *Shigella flexneri* | 4,609,642 | 50.46 | 163 | 245 | JBSVDX000000000 |
| DAK02031 | Dakar | Human Stool | 10 | F | *Shigella flexneri* | 4,374,695 | 50.50 | 155 | 6918 | JBSVDY000000000 |
| DAK02034 | Dakar | Human Stool | 1.7 | M | *Shigella flexneri* | 4,513,160 | 50.50 | 153 | 245 | JBSVDZ000000000 |
| DAK02084 | Dakar | Human Stool | 23 | F | *Shigella flexneri* | 4,528,028 | 50.45 | 119 | 245 | JBSVEC000000000 |
| DAK03008 | Dakar | Human Stool | 8.4 | M | *Shigella flexneri* | 4,472,541 | 50.73 | 160 | 145 | JBSVED000000000 |
| DAK03009 | Dakar | Human Stool | 3.5 | F | *Shigella flexneri* | 4,461,913 | 50.48 | 160 | 245 | JBSVEE000000000 |
| DAK01014 | Dakar | Human Stool | 1.9 | F | *Shigella sonnei* | 4,586,069 | 50.48 | 173 | 152 | JBSVDP000000000 |
| DAK02010 | Dakar | Human Stool | 7.4 | M | *Shigella sonnei* | 4,590,191 | 50.50 | 137 | 152 | JBSVDQ000000000 |
| DAK02023 | Dakar | Human Stool | 4.6 | F | *Shigella sonnei* | 4,576,024 | 50.49 | 163 | 152 | JBSVDW000000000 |
| DAK02072 | Dakar | Human Stool | 2.7 | M | *Shigella sonnei* | 4,705,658 | 50.53 | 141 | 152 | JBSVEB000000000 |
| DAK03011 | Dakar | Human Stool | 2.8 | F | *Shigella sonnei* | 4,601,768 | 50.50 | 161 | 152 | JBSVEF000000000 |
| FAT07026 | Fatick | Human Stool | 45 | F | *Shigella flexneri* | 5,544,605 | 50.25 | 134 | 245 | JBSVEG000000000 |
| KAO05004 | Kaolack | Human Stool | 56 | M | *Shigella sonnei* | 4,583,510 | 50.49 | 159 | 152 | JBSVEH000000000 |
| KMR16022 | Louga | Human Stool | 1.8 | F | *Shigella sonnei* | 4,554,467 | 50.51 | 147 | 152 | JBSVEI000000000 |
| STL11028 | Saint-Louis | Human Stool | 3 | M | *Shigella boydii* | 4,343,724 | 50.63 | 163 | 243 | JBSVEJ000000000 |
| TAM13030 | Tambacounda | Human Stool | 19 | F | *Shigella boydii* | 4,574,898 | 50.47 | 166 | 152 | JBSVEK000000000 |
